# Cobain, Hemingway, Sinatra and me

**DOI:** 10.7554/eLife.101618

**Published:** 2024-07-31

**Authors:** Lydia Krasilnikova

**Affiliations:** 1 Broad Institute of MIT and Harvard Cambridge United States

**Keywords:** sparks of change, being a scientist, bipolar disorder, mental health, research culture

## Abstract

A postdoc explains how being medicated for bipolar changed her life and the way she conducts research.

I wake up in the morning and start my day with lamotrigine, a mood stabilizer; I end it with cariprazine, an antipsychotic. In between, I study infectious disease outbreaks as a postdoc at the Broad Institute of MIT and Harvard. I write, I draw, I spend time with my loved ones, and all the other things that people generally do.

I'm bipolar, closer to I than to II, with psychosis, potentially schizoaffective. You might have heard some of these words before; for me, as for many others, they meant I was increasingly spending my life trapped in either a state of mania or depression.

My manic episodes would last about a month and a half and were characterized by intense productivity, creativity, confidence, conflict, and euphoria. I didn’t need sleep, food, or to be around people; think ‘Flip Me Upside Down’ by The Wombats, ‘Bad Habits’ by Ed Sheeran, and ‘Roadrunner’ by The Modern Lovers.

Euphoria is the best part of mania. Colors are actually brighter, music is actually better, and the secrets of the world are at your fingertips. I remember riding the Boston subway during my PhD, watching the Charles River roll by and feeling deeply connected to every person in my train car – thinking they were beautiful, wondering about their lives, wanting them all to wake up and feel the way I did, like a tiny spark in a tiny fire. In that moment I knew that if I died, the world would go on in a beautiful way.

There’s not as much to say about depression. I’d sleep up to 20 hours a day and either fade from life or simply disappear. Making food felt impossible; work felt impossible; a shower required spending half an hour on the floor just to hype myself up. My depression didn’t have any sadness to it, only a feeling that gravity had been turned up a notch or ten – think ‘Dying in a Hot Tub’ by Palaye Royale. If I died, I knew the world would go on, but the feeling wasn’t beautiful. Depression lasted as long as it took to recover from mania, often two or three months, at which point I’d flip back and the cycle would begin again.

I was diagnosed toward the end of grad school, in April 2022, after my therapist noticed temporal patterns in my moods. With this new lens to reflect on my life, I realized over the next few months that learning to work with and around bipolar had shaped my entire experience of higher education. In college, depression meant missing class and needing extensions (which I almost always got, luckily). Mania balanced out the impact of these periods, allowing me to forego sleep, learn quickly, and connect concepts easily.

In grad school, I was again productive enough when manic to compensate for nearly completely disappearing during my depressive episodes. At my peak, while working on the paper that represented the crowning project of my career so far, I slept every other day. Best of all, research ideas came easily. My manic self recorded them in detail, allowing my non-manic self to coast on those outlines. The flexibility that academia offers, its sometimes-fast, sometimes-slow pace, was a haven for me. Forgiving of bipolar. Rewarding of bipolar. How does anyone get through college without bipolar? How does anyone come up with research ideas or problem-solve without bipolar? How does anyone write a paper without bipolar?

But there isn’t much point to work if you don’t get to be alive. In the parts that feel good, it can be easy to lose sight of the fact that bipolar is a lot about death. Both mania and depression try to kill you directly. They also eat away at what makes life worth living, piece by piece, stealing a little bite with each cycle. In the end, it’s mania that did the most damage. In that heightened, impulsive, sometimes irritable state, the smallest slight could feel like an attack, triggering a desperate need to flee and defend myself. My mind played out a chorus of cruelties, delivered to me in the tone and mannerism of my mentors and my loved ones – teaching me, stealthily, not to trust the real people these voices belonged to. Waking up from a manic episode always included an apology tour and I watched my life deteriorate as the people I loved fell away. As soon as I was diagnosed, I asked for medication.

At first, I was worried that the meds would cause me to lose everything I had relied on so far in my work. And they did, in a way. The bursts of creative productivity are gone – I still miss them sometimes. I also miss euphoria. Worst of all, I need to sleep. I tried to pull an all-nighter shortly after starting treatment and not only was I not able to, I felt like my organs had been twisted around and scratched up. I felt like that for days – apparently that’s normal.

In the end, being medicated meant I had to change the way I do research. Instead of passionate bursts of productivity I work a little each day, amortizing my dreams, making slow progress. I prioritize and carefully plan my time. Turns out, that’s how things get done. To my surprise, I’ve been more productive on medication. I actually finish what I start, which used to be near-impossible as I jumped from thought to thought and failed to manage anxiety that led to procrastination. Now my research ideas emerge when I read, talk with my colleagues or even in my sleep; they aren’t any worse than when they used to come to me in bursts, faster than I could write them.

I can’t hear my mentors in my head anymore, harshly talking me through roadblocks at work. When I get stuck, I have to ask for help from the individuals the voices belong to – I find real people to be much more useful, and kinder too. And even with the antipsychotics, I got to keep the voice that dictated my writing and my ideas to me. It turned out to be what some would call an inner voice, and I can hear it better now that the other ones are gone.

Most importantly, the turmoil that characterized mania is gone, and so are the disappearances brought by depression. Without them, I can see how much these episodes were sinkholes for time, emotion, and productivity – depression in an obvious way, mania more stealthily. The internal and external conflicts it created cost me more than what I gained from its short bursts of productivity. I think that’s why I’m accomplishing more now. My mind is clearer, my interpersonal relationships more peaceful: I can actually focus on and enjoy my work and my life.

Many people I really admire were or may have been bipolar – from Ernest Hemingway to Jimi Hendrix, Frank Sinatra, Amy Winehouse, Kurt Cobain, Vincent van Gogh, and Carrie Fisher. You can count how many of them died young or by their own hand. Bipolar can be considered as a form of neurodiversity, but especially if left untreated, it is associated with a shorter life expectancy (13 years lower), increased levels of unemployment (up to 60%), and a suicide rate 10–30 times higher than for the general population (up to 20%). I hate saying this because it makes me feel ‘less-than’, but I’m very lucky to have a job, a partner, family members, and friends who have all stuck by my side. I’m very lucky to be alive.

Thirteen years ticked down between my first manic episode and my diagnosis, thirteen years during which I had no idea what bipolar was and the disease progressed exponentially – slowly, at first, and then, suddenly, very quickly, with increasingly intense episodes of mania and depression and nothing in between. I don’t think I had many manic episodes left in me before everything I loved about my life was gone.

Statistically, if you’re reading this, there’s roughly a 2% chance that you may see yourself in this article. If this is the case, know that medication could change things for the better for you, as it did for me. My past – my regrets, my accomplishments, the most beautiful feelings, the worst parts of myself – is ‘Roses’ by Carly Rae Jepsen; if I wanted to, on medication my life could be ‘Shop Vac’ by Jonathan Coulton (but without the sad parts). My meds, even the antipsychotics, haven’t robbed me of my creativity, my productivity, my inner voice, or my personality. I start my day with lamotrigine, I end it with cariprazine… And in between? I live, I work, and I get to be me.

